# Pneumatose kystique iléale révélée par un volvulus du grêle

**Published:** 2010-08-12

**Authors:** Bouhaddouti Hicham El, Abdelmalek, Benjelloun El Bachir, Majdoub Karim Ibn, Mazaz Khalid, Taleb Khalid Aït

**Affiliations:** 1 Service de chirurgie viscérale A, CHU Hassan II Fès, Maroc,; 2 Service de chirurgie viscérale B, CHU Hassan II Fès, Maroc

**Keywords:** Pneumatose, volvulus, bride, chirurgie

## Abstract

La pneumatose kystique intestinale est une pathologie rare qui se caractérise par la présence de kystes gazeux dans la paroi intestinale. Elle est asymptomatique ou pauci symptomatique, et le plus souvent découverte lors d’un examen d’imagerie ou d’endoscopie. Nous rapportons un cas de pneumatose iléale compliquée d’un volvulus sur bride chez un patient jamais opéré auparavant.

© Hicham El bouhaddouti et al. The Pan African Medical Journal - ISSN 1937-8688. This is an Open Access article distributed under the terms of the Creative Commons Attribution License (http://creativecommons.org/licenses/by/2.0), which permits unrestricted use, distribution, and reproduction in any medium, provided the original work is properly cited.

## Introduction

La pneumatose kystique intestinale (PKI) est définie comme la présence de kystes gazeux dans la paroi intestinale. Elle est rare mais sa fréquence est probablement sous-estimée.

Du Vernoi a fait la première description de PKI sur un cadavre humain en 1730 [1]. Jamart en 1979, a apporté d’importantes informations épidémiologiques à la suite d’une étude de 919 cas [2].

Cette affection bénigne est à distinguer de la pneumatose diffuse (ou emphysème intestinal), toujours secondaire, où le gaz est produit par la nécrose pariétale ou l’invasion bactérienne, comme dans l’entérocolite nécrosante, l’ischémie intestinale, les formes graves de colite pseudomembraneuse ou de colite cryptogénétique, et dont l’évolution est fulminante.

Il existe peu de données épidémiologiques car la PKI est généralement asymptomatique. Sur une série autopsique de 6553 sujets, deux cas seulement étaient recensés, soit une prévalence de 30/100000 [3].

## Patient et observation

Il s’agit d’un patient âgé de 51 ans, jamais opéré auparavant. Il est tabagique chronique et soufrant d’une broncho-pneumopathie chronique obstructive (BPCO) depuis 10 ans. Le malade présentait des douleurs abdominales atypiques intermittentes depuis un mois aggravées deux jours avant sa consultation aux urgences. Ces douleurs étaient accompagnées de vomissements alimentaires sans troubles de transit.

**Figure 1:**
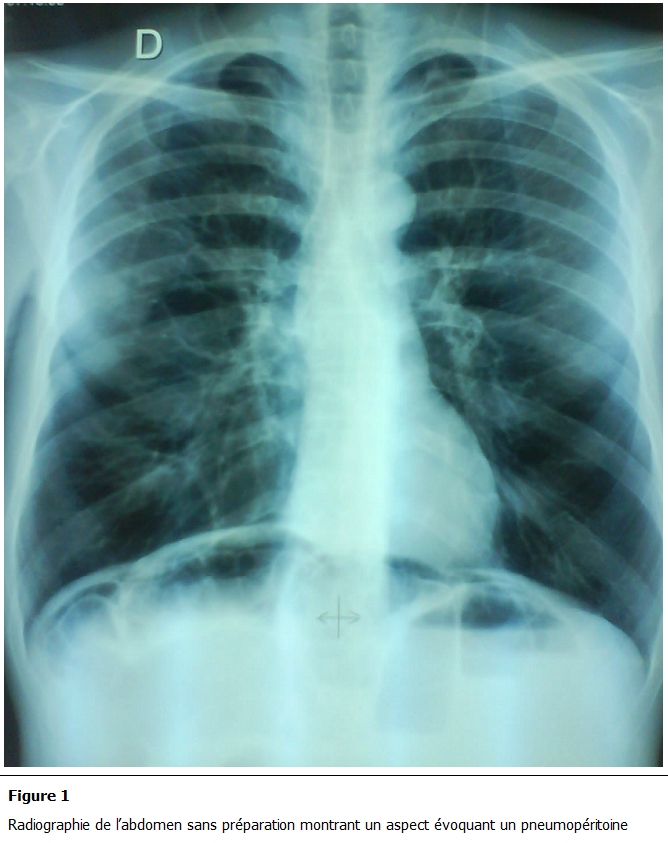
Radiographie de l’abdomen sans préparation montrant un aspect évoquant un pneumopéritoine

A l’examen, le patient était polypnéïque, avec un état hémodynamique stable et apyrétique. Il avait une distension abdominale modérée tympanique avec une sensibilité diffuse. La radiographie pulmonaire montrait un pneumopéritoine avec des niveaux hydro-aériques de type grêlique (figure 1). Une hyperleucocytose à 11200 éléments/mm³ a été retrouvée. Le patient a été opéré pour suspicion de péritonite par perforation digestive.

**Figure 2:**
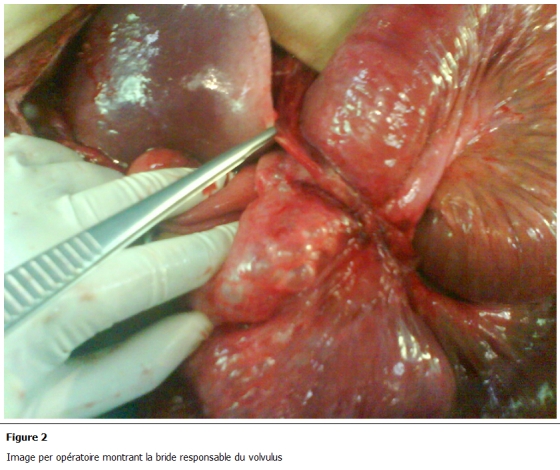
Image per opératoire montrant la bride responsable du volvulus

**Figure 3:**
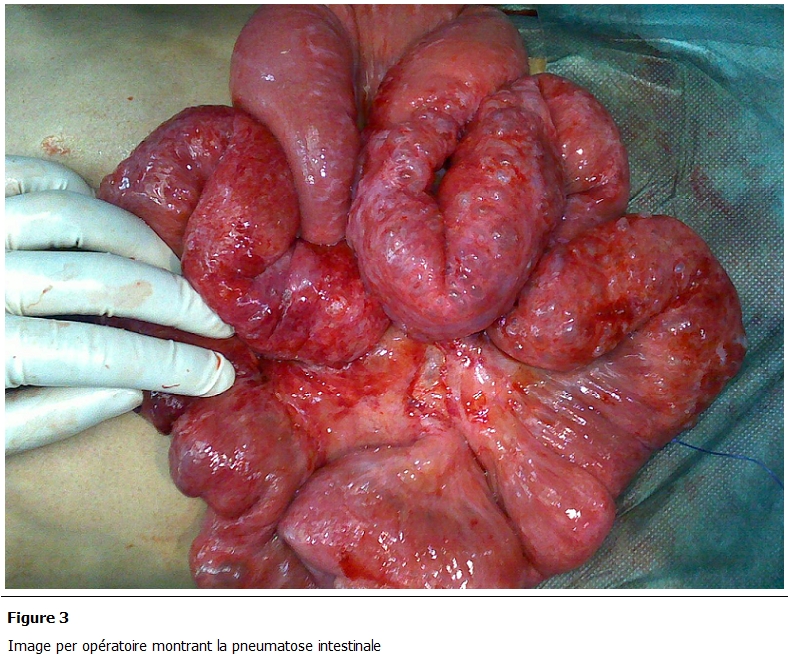
Image per opératoire montrant la pneumatose intestinale

L’exploration chirurgicale avait montré une distension grêlique secondaire à un volvulus iléal sur bride grêlo-hépatique (figure 2). L’intestin grêle était siège d’une pneumatose kystique diffuse étendue sur deux mètres à partir de 150 cm de l’angle de Treitz (figure 3). Le grêle a été dévolvulé après libération de la bride. La pneumatose kystique a été respectée. Les suites opératoires ont été simples. Le patient a quitté l’hôpital au 3 jour de son hospitalisation, a été revu à 1 mois puis à 3 mois, il est asymptomatique.

## Discussion

La PKI est caractérisée par des kystes qui siègent en sous-muqueux ou en sous-séreux de l’œsophage au rectum. Ils mesurent de quelques millimètres à plusieurs centimètres. Ces kystes sont disposés de façon segmentaire ou diffuse sur l’intestin, et leur étendue peut atteindre plusieurs mètres, elle est parfois sans rapport avec le site de la lésion causale. Les atteintes isolées du grêle sont les plus fréquentes (42%) suivies du côlon (32%); elles sont plus fréquentes que l’atteinte mixte ou extra-intestinale. Dans l’atteinte colique, le sigmoïde est concerné dans 70 % des cas, suivi du côlon descendant dans 40 % des cas, et des autres segments dans 15 à 25 % des cas, dont 10 % pour le cæcum [2].

Les PKI sont généralement sous-muqueux dans le côlon, prenant l’aspect de nodules polypoïdes sessiles, ils sont plus souvent sous-séreux dans l’intestin grêle, revêtant la forme de bulles en grappes de raisin, et se situent alors surtout sur le bord mésentérique [2]. La paroi des kystes est parfois très fine et peut être rompue, soit spontanément, soit après une biopsie endoscopique provoquant un véritable pneumopéritoine [4-6].

Dans 85 % des cas, la PKI est secondaire ou associée à d’autres pathologies gastro-intestinales (maladie inflammatoire intestinale, ulcère gastroduodénal, sténose pylorique, traumatisme abdominal) ou extra gastro-intestinales (broncho-pneumopathie chronique obstructive, cardiopathies, mucoviscidose, lupus, périartérite noueuse); les formes primitives ne représentent que 15 % des cas rapportés [4,5]. Chez notre patient, nous avons conclu à une pneumatose kystique liée à sa BPCO.

De nombreuses théories ont été proposées afin d’expliquer la PKI. Actuellement la pathogénie la plus probable associe les théories mécanique et bactérienne : association de lésions muqueuses, d’une augmentation de la pression intraluminale digestive permettant à des bactéries anaérobies (productrices d’hydrogène) de pénétrer dans la paroi intestinale. Le mécanisme princeps de cette pathologie est la brèche muqueuse qui paraît indispensable [7-9]. Pour d’autres, la pneumatose serait expliquée par un déficit en bactéries réduisant l’hydrogène : bactéries méthanogènes [9].

La PKI est généralement pauci-symptomatique. La plupart des auteurs rapportent des signes non spécifiques dans 30 % des cas : diarrhée, selles sanglantes ou glaireuses, météorisme, vomissements, constipation, ténesme [2]. Le météorisme abdominal est retrouvé dans 38 % des cas dans la série de Jamart [2]; l’occlusion luminale liée aux kystes peut être responsable de troubles du transit. Certaines complications liées au volume kystique ont été décrites : volvulus, invagination, perforation, hémorragie [10]. Elles sont rares (3 %) nécessitant alors des résections intestinales segmentaires [10].

La radiographie de l’abdomen sans préparation montre souvent un pneumopéritoine qui est dû à la rupture de kystes sous-séreux dans la cavité péritonéale. La pneumatose kystique est la première cause de pneumopéritoine sans perforation digestive. Celui-ci est présent dans 15 % des atteintes de l’intestin grêle et dans 2 % des atteintes coliques [7,8]. Les kystes sont mieux visibles sur la paroi colique. Ils se traduisent classiquement par des images aériques accolées entre elles et superposées à la clarté colique formant la classique image en « grappe de raisin » [7,11]

La tomodensitométrie avec opacification intestinale possède une bonne précision diagnostique [12]. Elle révèle des images de densité gazeuse dans la paroi digestive, mieux visibles en section transversale et en fenêtre pulmonaire [13,14]. L’association à un pneumopéritoine asymptomatique est quasi pathognomonique [2]. On a décrit un aspect échographique associant un amincissement de la paroi intestinale et des échos avec ombre acoustique, réalisant le «signe de l’aurore» [15]. Il existe un critère diagnostique important qui est l’absence d’aéroportie (à la différence des gangrènes intestinales) à la tomodensitométrie ou l’échographie [16].

En endoscopie, les kystes correspondent à de larges polypes sessiles hémisphériques, recouverts d’une muqueuse pâle et transparente, parfois ulcérée. Typiquement, on obtient l’affaissement du kyste à la ponction ou la biopsie avec un bruit d’éclatement [17].

Le traitement est le plus souvent médical. Son but est de réduire ou faire disparaître les kystes en en réduisant les bactéries anaérobies qui en sont à l’origine. L’antibiothérapie anti-anaérobie par le métronidazole est souvent efficace [8,11] mais d’autres antibiotiques comme l’ampicilline ou les fluoroquinolones ont permis d’obtenir de bons résultats [7]. L’oxygénothérapie hyperbare est utilisée pour son pouvoir anti-anaérobie et pour sa capacité à effondrer les kystes en favorisant les échanges avec le sang [8,18]. D’autres thérapeutiques telles que l’octréotide ou les fenestrations endoscopiques ont été utilisées avec des résultats variables [8]. Le traitement chirurgical est indiqué en cas de complications et en cas de symptomatologie rebelle au traitement médical [8,18]. Il consiste à réséquer le segment intestinal atteint par laparotomie ou encore mieux par laparoscopie. Ce dernier abord est préféré du fait de la bénignité de la pathologie et des conditions locales favorables (absence d’inflammation, absence d’adhérence imputable à cette pathologie).

## Conclusion

La PKI est une affection rare généralement bénigne sans symptomatologie spécifique. Son diagnostic est fondé sur les données de l’imagerie. Elle est souvent suspectée sur les radiographies de l’abdomen sans préparation et l’échographie. La TDM permet de confirmer le diagnostic, de préciser l’extension et de rechercher une éventuelle pathologie sous jacente. Les pneumatoses kystiques non régressives malgré un traitement médical bien conduit ou en cas de complication constituent une bonne indication au traitement chirurgical.

## Conflit d’intérêts

Les auteurs ne déclarent aucun conflit d’intérêt.

## Contribution des auteurs

Hicham El Bouhaddouti, Abdelmalek Ousadden, Bachir Benjelloun et Khalid Aït Taleb ont opéré le patient et participe a la rédaction du manuscrit. Tous les auteurs ont lu et approuvé la version finale du manuscrit.
